# Understanding the adoption context of China’s digital currency electronic payment

**DOI:** 10.1186/s40854-023-00467-5

**Published:** 2023-03-02

**Authors:** Huosong Xia, Yangmei Gao, Justin Zuopeng Zhang

**Affiliations:** 1 School of Management, Wuhan Textile University, Wuhan, 430073 China; 2 Research Center of Enterprise Decision Support, Key Research Institute of Humanities and Social Sciences in Universities of Hubei Province, Wuhan, 430073 China; 3 Research Institute of Management and Economics, Wuhan Textile University, Wuhan, 430073 China; 4 Department of Management, Coggin College of Business, University of North Florida, 1 UNF Dr, Jacksonville, FL 32224 USA

**Keywords:** DCEP, Push-pull mooring framework, Task-technology fit, Switch behavior, FinTech

## Abstract

Central banks worldwide have started researching and developing central bank digital currencies (CBDCs). In the digital economy context, concerns regarding the integrity, competition, and privacy of CBDC systems have also gradually emerged. Against this backdrop, this study aims to evaluate users’ willingness to use China’s digital currency electronic payment (DCEP) system, a digital payment and processing network, and its influencing factors by comprehensively considering and comparing the characteristics of cash and third-party payment services. Combining the push-pull-mooring framework (PPM) and task-technology fit (TTF) theory, we discuss the scenarios and mechanisms that may inspire users’ DCEP adoption intention through an empirical study. The results reveal that privacy concerns regarding the original payment methods and technology-task fitting level of DCEP positively impact users’ willingness to adopt DCEP. The technical characteristics of DCEP, users’ payment requirements, and government support positively affect users’ adoption intention by influencing the task-technology fitting degree of DCEP. Switching cost significantly and negatively impacts adoption intention, whereas relative advantage exhibits no significant effect. This research contributes to a better understanding of the factors that influence switching intentions and the actual use of DCEP, and provides policy guidance on promoting the efficiency and effectiveness of DCEP.

## Introduction

The integration of wireless communication, smartphones, and banking systems has created a digital payment ecosystem that is gradually replacing traditional cash payments. With technological advances, cash transactions have gradually evolved into noncash payments and governments worldwide are increasingly promoting digital payments, particularly in emerging economies (Gupta et al. 2020; Hung et al. 2021; Lonkani et al. 2020; Malaquias et al. 2021; Omigie et al. 2020; Wamba et al. 2021). Meanwhile, private digital currencies—such as Bitcoin and Libra, the super-sovereign digital currency issued by Facebook—have emerged consecutively, attracting widespread attention from central banks and all sectors of society. For instance, between 2019 and 2022, the digital currency electronic payment (DCEP) system was piloted and connected to Alipay and other platforms. By December 31, 2021, the number of DCEP pilot scenarios exceeded 8,085,100, with 261 million individual wallets opened and 87.565 billion Chinese yuan in transactions.

The concept of DCEP has two key aspects. First, DCEP is fiat money issued in digital form by the People’s Bank of China, operated by designated operating institutions, and offered to the public. Based on the broad account system, DCEP supports the function of loose coupling of bank accounts. Second, DCEP is equivalent to cash and coins, implying that it predominantly resides at monetary base (M0)—that is, cash in circulation, referring to the sum of the cash in the hands of enterprises, departments, and other units outside the banking system and the cash held by residents. M0 is closely related to consumption: The more it is, the more willing residents are to consume.

Developing countries are more active in the research, development, and promotion of central bank digital currencies (CBDCs), for which payment security, payment efficiency, and financial stability are the most important driving forces. As a CBDC, the DCEP provides a more convenient and secure payment method. Its issuance impacts the existing payment system, currency supply and demand, and economic and financial systems (Yao 2018). CBDCs exhibit unique advantages, such as settlement finality, liquidity, and integrity. The ultimate benefits of adopting new payment technologies depend on the competitive structure and data-management arrangements of the payment system. Digital currencies should be designed with public interest in mind, and CBDCs and open platforms should create virtuous cycles that favor greater access, lower costs, and better services (BIS 2021).

Digital and contactless payments accelerated owing to COVID-19. As a leader in the field of QR code payments, the Chinese market has cultivated two mature QR code payment methods—specifically, independent and dependent. Simultaneously, one of the most innovative and growing payment technologies is based on biometrics. American Express, Visa, and BNP Paribas have developed payment systems based on biometric technology. However, in developed regions, such as North America and Europe, the adoption level of these technologies is still insufficient (Liébana-Cabanillas et al. 2022). A payment system based on biometric technology can guarantee certain payment security because users are uniquely identified, but service providers must ensure users’ trust. Compared with cash, DCEP affects the privacy of user payment data, similar to the issues associated with mobile payments (Xia et al. 2022). However, unlike mobile payment platforms, the central bank—as a non-commercial entity—has no motivation to use private transaction data and can credibly commit to protect users’ data better than private banks or digital platforms, thus effectively addressing personal privacy issues and avoiding potential price discrimination against consumers (Auer et al. 2022). However, these advantages are based on the premise of assured data protection and public acceptance of DCEP.

DCEP benefits users with payment security, transaction costs, and payment efficiency. Additionally, DCEP’s unique monetary qualities, such as anonymity control, anti-counterfeiting trait, traceability, and programmability, also contribute to its efficacy in monetary policy and financial stability maintenance. However, with numerous competitive applications, DCEP cannot entirely replace existing payment instruments. Simultaneously, the promotion of legal digital currency faces social and technical resistance, as well as the impact of other digital currencies. Hence, whether DCEP can be widely used and accepted by the market and the public depends on the applicability of its application scenarios, as presented in Table 1. Under different national backgrounds, digital fiat currencies precipitate various user benefits. CBDCs may be more challenging to be adopted in developing countries than in developed countries because of the scale of the informal economy and anonymity of cash that allows users to hide their transaction histories in developing countries (Oh and Zhang 2022). The informal economy is an alternative source of growth and a buffer for the business cycle, but it is also characterized by low productivity, inadequate social protection, and financial exclusion. For emerging economies, CBDCs can cover a broader tax base by limiting tax evasion and other illicit activities, thus improving financial inclusion and overall economic mobility, offering formal electronic payment options for the unbanked, and facilitating the formalization of a long-term virtuous business cycle. These benefits can help alleviate the cost and complexity of payment system monopolies and fragmentation. For example, in China, the switching barriers and transaction fees between different mobile payment platforms could make DCEP a viable choice to match varying transaction scenarios, boosting its acceptance by consumers and merchants. In other economies, their existing payment systems are either already sufficiently robust or can be easily amended through additional improvements and upgrades. Nevertheless, currency substitution risks and other spillover effects associated with the CBDCs of large economies cannot be underestimated. The DCEP promises to address these issues; however, it must exhibit significant technological improvements compared to the existing payment systems (Soffer and Abir 2022).

**Table 1:** Context for DCEP

Users	Context for DCEP
Consumers	DCEP will further combine with the attributes of smart contracts to realize its functions for targeted crowds, scenarios, and usage which evolve from domestic to cross-border payment, urban to rural payment, and small to large payment
Business	From consumption application to production, the DCEP services will be extended gradually, with real-time payment service and no handling fee
Government	DCEP application will gradually cover various government service processes concerning social security, public accumulation fund, taxation, finance, and justice

Current studies related to mobile payment have predominantly focused on initial adoption and continuous usage behavior (Chin et al. 2020; Gupta et al. 2020; Humbani and Wiese 2019; Patil et al. 2020; Seth et al. 2020; Shao et al. 2019; Yuan et al. 2019), with a lack of attention to users’ switching behavior (Hu et al. 2021). As the issuing bodies of CBDC, banks also pay greater attention to fintech’s role in improving customer satisfaction (Kou et al. 2021a). Owing to the characteristics of DCEP and its advantages over other payment methods, it is suitable for exploring users’ switching behavior for specific DCEP payment scenarios (Gong et al. 2022).

As the basis for studying the mechanisms of switching behavior, the push-pull-mooring (PPM) model includes push factors that drive users away from existing services, pull factors that attract users to alternative services, and mooring factors that hinder or promote transfer behavior (Tang and Chen 2020; Loh et al. 2020). Prior studies have generally considered dissatisfaction, substitution attraction, and switching costs as push, pull, and mooring factors; and explored the switching behaviors of payment instruments impacted by the original habits of payment instruments, perceived security and operability, product involvement, and consumers’ attitudes (Lee 2019; Liu et al. 2021; van der Cruijsen and van der Horst 2019). However, the specific situational variables affecting users’ switching behavior have not been entirely determined. For example, existing mobile payment service providers require users to disclose highly confidential personal information to complete transaction processes, wherein privacy issues may either be a driving factor for switching or a mooring factor for continuous usage (Yang et al. 2019).

Against this backdrop, considering that DCEP is still in its pilot and promotion stages, this study aims to investigate users’ willingness to switch to DCEP from the current commonly adopted payment methods through the PPM framework and task-technology fit (TTF) theory by exploring the DCEP payment method’s advantages and application scenarios. Specifically, we aim to explore the following research questions:

- **RQ1** *What characteristics of the use context of DCEP are attractive to users?*
- **RQ2** *What is the relationship between the characteristics of DCEP, user payment requirements, and user concerns compared to other payment methods?*
- **RQ3** *How do the task-technology fitness and relative advantage of DCEP affect users’ willingness to adopt DCEP?*

## Theoretical backgrounds

### CBDC and DCEP

DCEP is an application of CBDC, issued digitally by the central bank to replace or supplement physical currency. The goal of CBDC design depends on the country’s national conditions, background, economic development level, and different development goals, which will lead to various design schemes for different CBDCs (Radic et al. 2021). The European Central Bank, Bank of Japan, Bank of England, and People’s Bank of China have all initiated pilot work for their respective CBDCs.

**Fig. 1:**
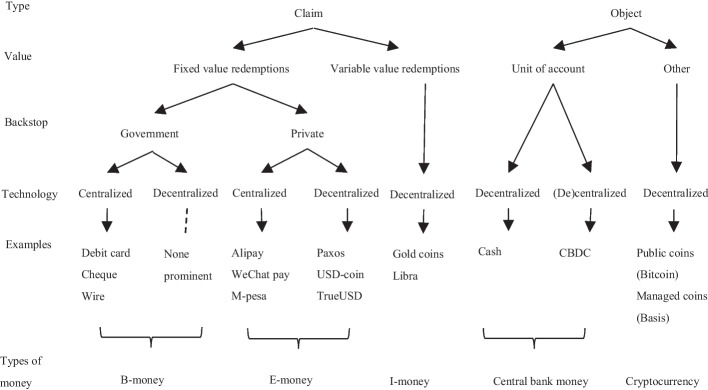
Money tree

The goals of CBDCs issued by various countries are predominantly to improve the efficiency and security of domestic and international payments and settlements, enhance financial inclusiveness, adapt to the development trend of a cashless society, enrich monetary policy tools, and combat with illegal and criminal behaviors (Kumhof and Noone 2021). Central bank digital currencies are more stable than private digital currencies because governments, which are effectively international organizations, have a higher degree of supervision. If electronic money is defined as all money existing in digital form, CBDC can be considered a particular type of electronic money (Hong et al. 2018). The difference is that CBDC is the direct liability of the central bank, whereas the issuing authorities of electronic money are commercial banks and other institutions that have obtained financial business licenses. Compared with cash, CBDC can reduce the incidence of crime, bribery, and fraud with lower operating costs. As a credit-based currency by value and a cryptocurrency by technology, CBDC can transform all aspects of the monetary system because it can benefit consumers as a cost-free medium of exchange, a store of value, and a stable unit of account. In the application scenario, CBDC is a smart currency which aims to deal with the challenges posed by digital currency. According to the financial report of science and technology released by the IMF that established the digital currency money tree structure (Adrian and Mancini-Griffoli 2021), we can divide digital currency by payment method, value stability, endorsement institution, and clearing technology into central bank money, b-money, e-money, investment money, and cryptocurrency (see Fig. 1).

DCEP has the following four major functions: QR code payment, remittance, receipt, and proximity payment based on Near Field Communication (Chabbi et al. 2020; Loh et al. 2022). DCEP’s controllable anonymity function is traceable, but compared with Alipay and WeChat, it has a lower risk of transaction data privacy disclosure.

In 2020, the Bank for International Settlements published the basic principles central banks should follow in the development of CBDC (Central bank digital currencies: foundational principles and core features). First, the form of money that the central bank provides should not interfere with its ability to execute its responsibilities to maintain monetary stability (Wilkins 2022). For example, the CBDC should allow the public to use different forms of currency interchangeably. Furthermore, different types of central bank money and existing cash, reserves, or settlement accounts should complement each other and coexist with private funds, such as commercial bank accounts. Central banks should continue providing cash as long as sufficient public demand prevails. Second, when payment services are provided, central banks have a responsibility to create a safe, efficient, and user-friendly system, whereas private economic entities should be free to decide the methods of payment to use in their transactions (Andolfatto 2021). As DCEP is a public product provided by the People’s Bank of China for the domestic retail payment market with a natural endowment, its primary function is fulfilling the diversified payment needs of all kinds of people at all levels. Based on the credit endorsed by the country, DCEP must be more deeply connected with the payment requirements of enterprises and companies, and provide a safe and controllable payment system that can fully maintain financial stability while coexisting with other payment systems (see Table 2).

**Table 2:** Characteristics of DCEP

Traits	Explanation
Legality	The DCEP is issued and managed by the People’s Bank of China
Technology characteristics	Circulation, storage, offline transaction, controllable anonymity, unforgettably, non-repeatable transaction, and non-repudiation
Position	The DCEP is mainly positioned as cash in circulation (M0), a public product provided by the central bank to the public, and will not charge for services such as fund intercourse
Currency issuance	A two-tier operating system: “Central Bank-Commercial Bank” dual system
Core system	Digital currency-issuing database, digital currency commercial bank database, certification center, registration center, and big data analysis center
Monetary Account	Broad Account System: Anything that can uniquely lock a personal identity can become an account
Wallet opening	Loosely coupled accounts: A DCEP wallet can be launched without a bank account
Currency anonymity	Anonymous payment within a certain amount
Monetary credit	National Credit Endorsement

The DCEP aims to respond to people's demands for a personal payment system that combines stability, security, data privacy, and ease of use (Bhaskar et al. 2022; Dow 2019; Tong and Jiayou 2021; Hofmann 2020). A sound network security governance mechanism is a critical condition for CBDC issuance and promotion. It is also an urgent topic for countries to evaluate their computational costs to measure their feasibility and practicability while maintaining security attributes, such as anonymity, liability, and traceability (Liu et al. 2022). The relevant literature reflects the concern for a stable and secure cashless society in the future and discusses the policy implications of digital currency, such as the pros and cons of a cashless society; the prospects, motivations, and challenges for its implementation; and subsequent social responses (Li et al. 2022; Náñez Alonso et al. 2020).

In the context of global economic and structural diversity, the promotion of DCEP faces the challenges of legislation, costs, optimal monetary policy, and other issues. The promotion of digital fiat currency is confronted with international challenges (Minesso et al. 2022). Economic, social, political, business ethics, and environmental factors influence the application of CBDC, which will also be influenced by CBDC (Chen and Siklos 2022; Ding et al. 2022). Meanwhile, relevant studies discuss the influence of CBDC by regulators and media on financial markets (Wang et al. 2022a, b).

However, CBDCs’ real impact on banking needs to be demonstrated through their development and will also vary with their liquidity levels. Although the pace of CBDC advancement varies by country, it is necessary for society’s digital transformation. The mobile payment industry is flexible and innovative, which may cause users to disregard the advantages of CBDC. A combination of mobile payment tools may be the optimal way forward (Náñez Alonso et al. 2021). A research trend in digital currency addresses how digital currency changes consumers’ choice of payment instruments and currency demands (Shi and Sun 2020). With technological progress, available payment methods have expanded from cash to checks, credit cards, debit cards, and direct transfers, thus increasing the need to consider payment portfolios. However, only a few studies have analyzed the characteristics of each payment option. Exploring these distinctive features is conducive to understanding people’s preferences and changes when choosing payment services, thus contributing to determining the demand for corresponding payment methods (Son et al. 2022).

### Push-pull-mooring framework and switching behaviors

With the pilot tests of DCEP, its application scenarios are also expanding and becoming enriched. DCEP services can be provided (1) for customers, namely, consumption scenarios involving personal payments; (2) for business, that is, enterprise business scenes, such as enterprise procurement and salary payment; and (3) for cross-border payment. Currently, the Digital Currency Research Institute of the People’s Bank of China has launched the “Multilateral Central Bank Digital Currency Bridge” project to explore the application of DCEP in cross-chain payments. Since December 2019, under the guidance of the People’s Bank of China, banks such as the Industrial and Commercial Bank of China have been committed to targeting the following application contexts: inclusive finance, campus culture, tourism, government service payment, farmers, and agricultural support.

DCEP and non-bank payment institutions will not compete with or replace each other, nor will they completely substitute cash. DCEP is equal to money and is a tool that provides users with more diversified payment options to perform functions as infrastructure and a carrier of digital fiat money as third-party payment does, which will continue participating in the circulation process of DCEP as the financial infrastructure of DCEP wallet.

The PPM framework divides the factors affecting people’s migration from one area to another into the following three aspects: push, pull, and mooring. This model was first used to study population migration and consumer switching behavior. This model not only examines the pushing and pulling effects of relevant factors on users’ switching behaviors from the perspectives of original products/services to alternative products/services, but also examines the mooring effect at individual and social levels (Fan et al. 2021; Zhang et al. 2021).

The pushing factor refers to the factor that pushes users of a product away from the original product; the pulling factor refers to the factor that promotes users to use the new product; and the mooring factor refers to the personal or social factor that can hinder or promote users’ transfer behavior. According to the PPM framework, migration or switching behavior results from the interaction of push-pull effects and is interfered with by mooring effects (Handarkho and Harjoseputro 2019).

Common factors influencing users’ switching behavior include quality, satisfaction, value, trust, commitment, price perception, alternative attitudes, social impacts, switching costs, previous switching behavior, and diversification tendencies (Kesharwani et al. 2021; Li 2021; Rahman et al. 2020; Rouibah et al. 2021; Sampaio et al. 2021; Shiau et al. 2021; ZareRavasan and Krčál 2021). Digital currency and electronic payment research focusing on consumers’ choices of payment tools is increasing (Wu et al. 2022). Previous studies have revealed the following two challenging steps in guiding payment behavior: (1) changing payment preferences and (2) the actual payment choice to match new payment preferences (van der Cruijsen and van der Horst 2019). The original PPM model primarily focuses on users’ switching behavior for the same type of applications. However, due to the lightweight mobile application design, modest learning curves, and low switching costs, numerous users tend to exhibit partial IT switching behavior, which refers to entirely or partially replacing an IT product or service with a substitute that meets similar needs, analogous to human migration behavior (Hu et al. 2021; Mu and Lee 2022). Unlike the previous “one-or-another” selection option, users choose the “multiple-in-use” style based on different payment contexts and requirements.

With technological progress, available payment methods have expanded from cash to checks, credit cards, debit cards, and direct transfers, increasing the need to consider payment portfolios. However, only a few studies have analyzed the characteristics of each payment asset. Exploring these features is conducive to understanding people’s preferences and changes when choosing payment services, and identifying the distinctive features of each payment method is essential in determining the demand for the use of corresponding payment methods (Son et al. 2022). According to the analysis of the monetary tree, DCEP is essentially different from other payment applications, and its services also exhibit different characteristics, especially in terms of data protection. By combining the PPM framework and TTF theory, this study analyzes whether DCEP can meet the same or even more comprehensive payment needs while reflecting its relative advantages with third-party payment services, especially from the perspective of business and government payment service requirements.

## Hypotheses development and model design

### Pull factors

Mobile commerce offers convenient services. However, security and privacy have always been the main concerns for most users (Tang et al. 2020; Wang et al. 2020). For mobile payment service providers, unifying anonymity, security, and efficiency is challenging (Cao and Zhu 2019). Third-party payment platforms usually require users to submit different private information, but users rarely understand how their personal information will be used. The traceback of DCEP requires payment and clearing systems to report institutions, individuals, and clearing checkpoints that DCEP passes in the circulation and clearing process, as well as building a log system that is recorded by the central government and covers the entire life cycle of DCEP. Previous studies have suggested that, as perceived privacy issues decrease, perceived trust increases and perceived risk decreases, while the perceived risk is a deterrent to mobile payment adoption. The decentralization of DCEP and characteristics of small anonymous payments may reduce users’ perceived privacy risks in payments (Chin et al. 2020). Therefore, we propose the following hypothesis:

#### **H1a**

Privacy concerns pertaining to original payment methods positively impact users’ willingness to use DCEP.

The literature on market-based e-commerce recognizes the importance of institutional trust. Platforms act as intermediaries in the exchange of services between customers and service providers. A trusted platform can implement necessary actions to provide a reliable and secure trading environment, thus reducing customers’ risk perception and leading to continuous use behavior (Kondrateva et al. 2020). The effectiveness of users’ perception of third-party hosting services—such as PayPal, Alipay, and WeChat Pay—is reflected in whether users can be protected from potential risks and illegal behaviors through general institutional mechanisms, regulations and rules, privacy and data security, minimum insurance requirements, and regular security checks (Lu et al. 2021).

#### **H1b**

Privacy concerns regarding the original payment methods positively impact the relative advantages of DCEP and, thus, positively impact users’ willingness to use DCEP.

DCEP has three specific advantages as follows: First, it is a national legal tender with a high security level. Second, DCEP can transfer value without relying on bank accounts, support offline transactions, and retain the characteristics of payment and settlement. Third, DCEP supports controllable anonymity, which is conducive to the protection of personal privacy and user security. In the use of new technologies, the sense of insecurity refers to consumers’ suspicion of new technologies, while the sense of discomfort refers to consumers’ ability to use and control new technologies, both of which slow down users’ willingness to adopt new technologies (Chen et al. 2021; Chu et al. 2022; Humbani and Wiese 2019). Among the design principles of the DCEP system, security is the basis for other principles. As a central bank, carefully considering the security and disaster tolerance of its technical and business systems is necessary (Pal et al. 2021). A completely anonymous CBDC design may seriously affect the integrity of payment systems, leading to illegal transactions or money laundering. DCEP is exploring how to protect consumers’ payment privacy while providing more convenient and secure payment services. DCEP adopts a consumer privacy protection mechanism that is anonymous for small payments and traceable for large amounts according to the law while establishing an information isolation mechanism. The digital wallet inquiry, freezing, and deduction conditions are still under investigation. The subsequent step is improving the legal system of DCEP and establishing a corresponding punishment mechanism to ensure that a third party does not obtain the privacy of relevant transactions without legal authorization or disclosure. Hence, we propose the following hypothesis:

#### **H1c**

DCEP security perception positively impacts users’ willingness to use DCEP.

Security and risk perception are major concerns in electronic payment. The risks associated with the new technology originated from data and privacy generated in transactions conducted for themselves. If innovative technologies succeed, they must control the security of their new payment system. Only when people perceive that they can control the security of mobile payment systems do they successfully use such technologies (de Luna et al. 2019). The customer authentication system for electronic payment includes (1) things that only users know (e.g., passwords), (2) things owned by the customer (e.g., the communication code received and verified by a user’s mobile phone), and (3) things inherent in a user (e.g., iris or fingerprint). From users’ passwords to their biometric data, developing payment systems can ensure security and convenience, but privacy and security have also become hidden dangers, especially for China's rapidly developing third-party payment industry. The risks precipitated by operational accidents, such as violations of laws and regulations and network attacks faced by third-party payment platforms, cannot be underestimated. The limited data disclosed by the official and private sectors has challenged risk assessment (Yao and Li 2022). Regarding legal risk, speculation, website vulnerabilities, and hacker attacks, DCEP faces more severe tests, but it also has advantages that are difficult to imitate. Based on this rationale, we formulated the following hypothesis:

#### **H1d**

Perceived security of DCEP positively affects its relative advantages, thus, positively impacting users’ willingness to use DCEP.

According to the diffusion theory of innovation, users’ innovation adoption behavior is influenced by relative advantage, compatibility, testability, observability, and complexity (Lin et al. 2020). Relative advantage refers to the degree to which an innovation is considered better than the product or service it replaces (Law et al. 2021; Li and Wang 2021; Sultana et al. 2021; Yang and Yi 2021); the higher individuals perceive the relative advantages of innovation, the more likely they will be to adopt it. When an innovative technology is more convenient, efficient, and popular than the original product or service, implying its relative advantage, people will be positively willing to use the innovation (Lin et al. 2021; Sun et al. 2021). Hence, we propose the following hypothesis:

#### **H1e**

The relative advantage of DCEP positively affects users’ willingness to use DCEP.

### Push factors

Information technology services should be well suited to the tasks that they support to implement and positively influence user performance. Technology-task fit aims to bridge the gap between task requirements and technical attributes. In the TTF model, technical and task characteristics affect users’ perceptions of technology-task fit and willingness to use. According to TTF theory, adoption behavior depends partially on how well a technology fits the requirements of a particular task. If a technology matches the task it supports, it will be adopted (Franque et al. 2022). The factors determining the success of technology-task-fit and system applications include the characteristics of tasks, technologies, and individual users. Accordingly, we formulated the following hypothesis:

#### **H2a**

Users’ perception of technology-task fit on DCEP positively affects users’ willingness to use DCEP.

In recent decades, various techniques have been closely combined with information and communication technology to provide an effective and low-cost way to create a relative advantage and maintain customer loyalty (Pal et al. 2021). For example, an electronic banking platform can provide its customers with services available 24 h a day for account inquiries, transfer and payment to a third party, account checks, consultation, and deposit functions. User requirements for payment methods include fast transaction speed, tight control over service interaction, short waiting time, personalized service perception, and the convenience of obtaining services that are not limited by time or space (Chang et al. 2021; Sánchez-Torres et al. 2018; Wamba et al. 2021). The design scheme of the DCEP requires full consideration of its convenience, safety, and general characteristics (Yao 2018). The circulation system of the DCEP requires three levels to be harmonious and unified: technology method, mechanism design, and laws and regulations, which address problems of convenience and safety, privacy, antonymy, simplified trade links, technology integration, and innovation. Hence, the following two hypotheses are proposed:

#### **H2b**

The characteristics of users’ payment demands positively affect users’ technology-task matching perception and, thus, positively impact users’ willingness to use DCEP.

#### **H2c**

The technical characteristics of DCEP positively affect users’ perception of technology-task fitting and, thereafter, positively impact users’ willingness to use DCEP.

### Mooring factors

Status-quo preference theory suggests that people prefer staying where they are rather than change. Switching costs indicate that users are more inclined to maintain status quo when switching costs are higher than benefits (Pal et al. 2020). Some scholars have studied the influence of transfer cost on transfer intention from three perspectives—namely, procedure, financial, and relationship switching costs. The process switching cost includes learning and establishment costs. The economic switching cost includes loss of income and monetary costs. The relational switching cost includes personal and brand relationship loss costs (Sánchez-Torres et al. 2018). Our study concentrates on selection procedures and financial switching costs in a partial switching context. Thus, we propose the following hypothesis:

#### **H3a**

Switching cost negatively affects users’ willingness to use DCEP.

The government can play its part by enacting clear laws to ensure consumers have greater confidence in DCEP and helping retailers encourage user adoption. Government support is often considered in studies exploring the willingness to adopt technology or services (Chen et al. 2020). However, social factors have received minimal attention in recent studies on switching behavior. Based on the human-technology-organization framework for reference, the PPM framework is supplemented to explain and predict users’ willingness to switch from original payment methods to DCEP—to allow for exploration methods and countermeasures that promote users’ willingness to consider DCEP.

When trusted sources support technologies, users are drawn to them. Government support simplifies the technology adoption process by promoting the development of a technology base and acceptance of specific methods of use. Government support can intervene and guide the diffusion of technological innovation, and government regulations can isolate or hinder innovation adoption (Chen et al. 2020;Choudhuri et al. 2021; Sánchez-Torres et al. 2018). When the government provides or supports services, confidence and willingness to use them increase, and government interventions affect individuals’ risk-taking behavior, value creation, and sense of security. Hence, we propose the following hypothesis:

#### **H3b**

Government support positively affects users’ willingness to use DCEP.

Previous studies on the factors influencing the adoption of innovative technologies from multiple organizational perspectives have demonstrated that adoption decisions depend on organizational, technological, and individual factors. Cooperation between the government and financial institutions influences people’s sentiments and attitudes toward financial services (Qu et al. 2022). As a strong institution supporting banks, the government can convince people that DCEP is safe and that its operating system is ethical and effective. Hence, we propose the following hypothesis:

#### **H3c**

Government support can positively influence users’ technology-task fit perception and, subsequently, positively impact users’ willingness to use DCEP.

**Fig. 2:**
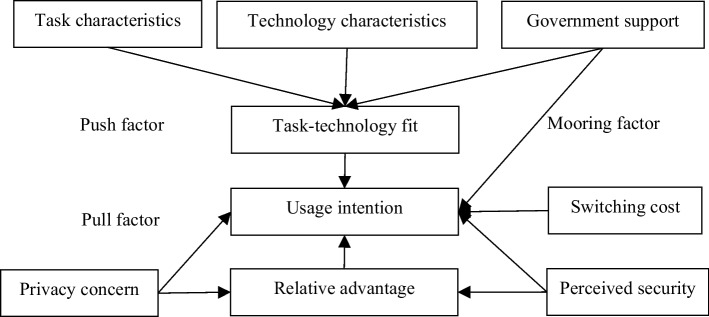
Research model

Figure 2 displays the research model by integrating all the constructs.

## Methodologies and model test

### Measurement and data

#### Measurement

In the questionnaire design, indicators used in previous scales were adopted as much as possible. The related variables in the model were modified considering the characteristics of various payment methods and DCEP.

#### Demographic characteristics of the samples

As this study aimed to investigate the characteristics of individual users and their behavioral intention to use DCEP, the questionnaire was designed for individuals with daily payment behaviors. The introduction of DCEP was added to the introduction section of the survey. DCEP is not a fictional novelty, and trials of DCEP basically cover the Yangtze River Delta and Pearl River Delta, Beijing-Tianjin-Hebei region, basically covered central, northeast, and northwest China. Various banks and government institutions have also promoted the use of DCEP to prevent fraud. The users’ most commonly used payment methods were first examined when the questionnaire was issued. In the pre-experiment phase, the questionnaire was revised thrice, based on the total time required and public feedback on any other problems encountered during the completion process.

The questionnaires were distributed and collected over a wide range of wenjuanxing.com, QQ, and WeChat moments by links and QR codes. The pre-experiment and formal collection of questionnaires lasted for two weeks, and cash rewards were provided to respondents who completed the questionnaires. After excluding invalid questionnaires based on test items and filling times, 372 valid questionnaires were obtained. Table 3 displays the descriptive statistics of the respondents’ basic information, including gender, age, and educational level.

**Table 3:** Demographic characteristics of the samples

Level	Frequency	Percentage	Accumulated %
Gender			
Male	194	52.2	52.2
Female	178	47.8	100.0
Age			
18–35 (including 18)	195	52.4	52.4
35–45 (including 35)	104	28.0	80.4
45–60 (including 45)	64	17.2	97.6
> 60	9	2.4	100.0
Level of education			
High school or below	33	8.9	8.9
Technical secondary school	118	31.7	40.6
3-year or 4-year college	176	47.3	87.9
Graduate school or higher	45	12.1	100.0
Original payment method			
Paper-based payment (notes, coins, cheques, promissory notes, money orders)	61	16.4	16.4
Card-based payment (bank card, prepaid card)	138	37.1	53.5
Net-based payment (card-free payment based on a bank account or third-party account by network terminal)	173	46.5	100.0

### Examining the model

#### Reliability and validity

The results of the confirmatory factor analysis (CFA) and correlation analysis are presented separately in Tables 4 and 5. Regarding reliability, the standardized factor load was greater than 0.6. The combined reliability CR value was greater than 0.8, and the AVE value of the average extraction variation was above the acceptable threshold value of 0.36. Therefore, the scale used in this study exhibited good convergence validity. Regarding discriminative validity, the square root AVE value of the latent variable was greater than its correlation coefficient with other variables, indicating that the scale used in this study had good discriminative validity. The variance inflation factors of all explanatory variables ranged from 1.011 to 1.141, which was less than the critical value of 10, indicating that the model did not exhibit an evident multicollinearity problem.

**Table 4:** The results of the confirmatory factor analysis (CFA)

Constructs	Items		Loadings	References
Task characteristic (TAC)	TAC1	I need to know and manage my financial accounts anytime and anywhere	0.833	Chen and Tan (2018)
	TAC2	I need to be able to transfer money to my financial account anytime and anywhere	0.856	
	TAC3	I need access to my financial account anytime, anywhere	0.811	
Technology characteristic (TEC)	TEC1	DCEP can offer services that are different from other payment applications	0.840	
	TEC2	DCEP can provide real-time payment services		
	TEC3	DCEP can provide secure payment services	0.817	
	TEC4	DCEP can provide fast payment services	0.848	
Task-technology fit (TTF)	TTF1	The functions of the DCEP in terms of payment are sufficient	0.847	
	TTF2	DCEP can provide unique services that meet users’ needs	0.824	
	TTF3	In general, the functions provided by DCEP are appropriate	0.808	
Privacy concern (PC.)	PC1	I am concerned that payment methods commonly used today may collect my personal information without telling me	0.860	Zhou et al. (2021)
	PC2	I am concerned about the potential misuse of personal information submitted to payment methods now commonly used	0.845	
	PC3	I worry that other people will be able to find my private information through the payment methods I now use	0.855	
	PC4	I am concerned about giving personal information to the currently common means of payment because the consequences are unpredictable	0.852	
Percirved security (PS.)	PS1	I believe that DCEP have a secure web for electronic transactions	0.814	Escobar-Rodríguez and Carvajal-Trujillo (2014)
	PS2	I consider that DCEP guarantees that my payment process is not interrupted and my transaction information is not lost	0.868	
	PS3	I consider that online banks keep and handle my personal information safely	0.827	
Relative advantage (RA.)	RA1	DCEP is more efficient than other methods of payment	Delete	Chen and Tan (2018)
	RA2	DCEP is easier to try than other payment methods	0.875	
	RA3	Compared with other payment methods, DCEP is subject to fewer time restrictions and space restrictions	0.843	
	RA4	Compared with other payment methods, DCEP is more reliable to use	0.862	
Switching cost (SC.)	SC1	It takes me some time to learn how to use DCEP	Delete	Kim and Kankanhalli (2009)
	SC2	I need to learn to use DCEP at a loss	0.904	
	SC3	It takes me some time to register and get familiar with the DCEP	0.894	
	SC4	I’m not sure the DCEP will bring me better products and services	0.892	
Government support (GS.)	GS1	Overall, I ink the government has policies that promote the use of DCEP	0.854	Sánchez-Torres et al. (2018)
	GS2	Overall, I think the government is promoting the development of DCEP	0.855	
	GS3	Overall, I think the government has a favorable legislation to use DCEP	0.837	
Usage intention (UI.)	UI1	I’d like to try using DCEP	0.827	
	UI2	It is more likely that I will try to use DCEP	0.868	
	UI3	I’m thinking about starting to try to understand and use DCEP	0.791

**Table 5:** Correlations and descriptive statistics

Variables	Mean	SD	α	AV	CR	1	2	3	4	5	6	7	8	9
1. Privacy concern	3.824	0.976	0.881	0.727	0.914	0.853								
2. Task characteristic	3.922	0.934	0.807	0.804	0.925	.142**	0.897							
3. Technology characteristic	3.959	0.915	0.810	0.740	0.895	.129*	0.086	0.860						
4. Relative advantage	3.908	0.962	0.840	0.720	0.885	0.101	.174**	.117*	0.849					
5. Task-technology fit	3.972	0.911	0.798	0.700	0.875	0.053	.196**	.186**	0.065	0.837				
6. Government support	3.881	0.986	0.826	0.698	0.874	0.096	.109*	.113*	.159**	.205**	0.835			
7. Perceived security	3.902	0.927	0.820	0.687	0.868	0.057	.168**	.270**	.139**	.187**	.166**	0.829		
8. Switching cost	3.466	1.221	0.884	0.695	0.872	− 0.004	− 0.014	− 0.034	− 0.091	0.001	− 0.047	− 0.046	0.834	
9. Usage intention	3.913	0.905	0.808	0.683	0.866	.168**	.195**	.144**	.128*	.216**	.128*	.213**	− .120*	0.826
VIF						1.044	1.100	1.122	1.078	1.117	1.090	1.141	1.011	

#### Common method variance

This study adopted the Harman single-factor test to conduct factor analysis on all questionnaire items. As illustrated in Table 6, the first principal component obtained without rotation explained 16.688% of the variation. Thus, homologous variance between measured variables would not affect the conclusion’s reliability.

**Table 6:** Total variance explained

Component	Initial Eigenvalues			Extraction sums of squared loadings		
	Total	% of variance	Cumulative %	Total	% of variance	Cumulative %
1	4.673	16.688	16.688	4.673	16.688	16.688
2	2.760	9.858	26.545	2.760	9.858	26.545
3	2.516	8.986	35.532	2.516	8.986	35.532
4	2.153	7.690	43.222	2.153	7.690	43.222
5	2.051	7.326	50.548	2.051	7.326	50.548
6	2.000	7.142	57.690	2.000	7.142	57.690
7	1.680	6.001	63.692	1.680	6.001	63.692
8	1.658	5.923	69.614	1.658	5.923	69.614
9	1.437	5.131	74.745	1.437	5.131	74.745
10	.627	2.241	76.986			
11	.526	1.877	78.863			
12	.499	1.781	80.644			
13	.484	1.728	82.372			
14	.463	1.653	84.025			
15	.439	1.569	85.594			
16	.416	1.485	87.079			
17	.401	1.431	88.510			
18	.374	1.335	89.846			
19	.357	1.274	91.120			
20	.350	1.250	92.370			
21	.326	1.164	93.535			
22	.311	1.109	94.644			
23	.286	1.020	95.664			
24	.279	.996	96.660			
25	.259	.926	97.586			
26	.242	.866	98.452			
27	.233	.833	99.285			
28	.200	.715	100.000			

#### Path analysis

A structural equation model was used to analyze the path. Table 7 illustrates that the selected indicators meet the standards of the reference table, according to which we consider the research model acceptable.

**Table 7:** Model fitting results

Simulation fit index	Statistics	Ideal range	Fitting situation
CMIN/DF	1.240	1–3	Good
RMSEA	0.025	< 0.050	Good
GFI	0.931	> 0.900	Good
AGFI	0.913	> 0.900	Good
IFI	0.982	> 0.900	Good
CFI	0.982	> 0.900	Good
NFI	0.914	> 0.900	Good
PGFI	0.738	> 0.500	Good
PNFI	0.779	> 0.500	Good

**Fig. 3:**
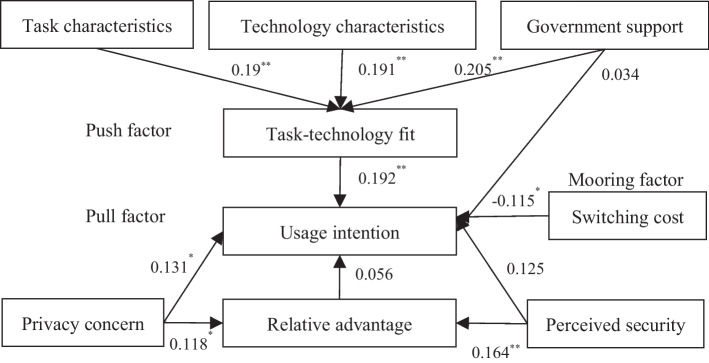
Hypothesis testing effect of the integrated model

AMOS 26.0 was used for path analysis to verify the research hypothesis in this study, and the comprehensive results are displayed in Fig. 3. We believe that this hypothesis is generally valid when P < 0.05. Based on the results of the path test, displayed in Table 8, it can be concluded that users’ privacy concerns about the current payment methods exhibited a significant positive impact on their willingness to use DCEP. Users’ perceptions of the technical characteristics of DCEP, demand characteristics of individual payment processes, and government’s support for the promotion of DCEP positively affected their technology-task fit perception. Users’ technology-task fit perception exhibited a significant positive impact on their willingness to use DCEP, while switching costs negatively impacted users’ DCEP adoption intention.

**Table 8:** Pathway analysis results

Pathway	Unstandardized coefficients	Standardized coefficients	SE	CR	P	Results
TAC → TTF	0.194	0.19	0.064	3.023	0.003	Supported
TEC → TTF	0.197	0.191	0.065	3.026	0.002	Supported
GS → TTF	0.199	0.205	0.062	3.226	0.001	Supported
PC → RA	0.121	0.118	0.061	1.972	0.049	Supported
PS → RA	0.193	0.164	0.073	2.645	0.008	Supported
TTF → UI	0.161	0.192	0.057	2.824	0.005	Supported
RA → UI	0.044	0.056	0.048	0.916	0.36	Not supported
GS → UI	0.028	0.034	0.052	0.528	0.598	Not supported
PC → UI	0.106	0.131	0.049	2.161	0.031	Supported
SC → UI	− 0.074	− 0.115	0.037	− 1.971	0.049	Supported
PS → UI	0.116	0.125	0.063	1.826	0.068	Not supported

From the perspective of the risk factors of fintech and push factors of the PPM framework, the hypothesis that the relative advantage positively impacts the willingness to use DCEP is not tenable. However, users’ privacy concerns regarding the currently commonly used means of payment would affect their perception of the relative advantage of DCEP. Users’ privacy concerns regarding commonly used payment methods positively impacted their willingness to use DCEP. After communicating with some interviewees, possible explanations for these outcomes are as follows: Currently, DCEP is still in the pilot stage, and users outside the pilot cities are still unfamiliar with DCEP and have not experienced its relative advantages. Some respondents believed there was minimal difference between DCEP and previous payment methods. The relative advantages of DCEP still need to be explored.

Using the AMOS 26.0, the Bootstrap method was used to test the existence of the mediation effect in the research model. The theoretical basis is that if there is no 0 between the upper and lower limits of the confidence interval of the mediation effect, the mediation effect exists and is significant. The results are presented in Table 9.

**Table 9:** Unstandardized Bootstrap mediation effect test

Pathway	Value	SE	Bias-corrected 95%CI			Percentile 95%CI			Results
			Lower	Upper	P	Lower	Upper	P	
indPC	0.005	0.009	− 0.004	0.036	0.204	− 0.011	0.025	0.527	Not supported
indPS	0.009	0.013	− 0.009	0.053	0.232	− 0.015	0.039	0.469	Not supported
indTAC	0.031	0.02	0.005	0.092	0.013	0.001	0.082	0.033	Supported
indTEC	0.032	0.02	0.005	0.085	0.011	0.003	0.078	0.024	Supported
indGS	0.032	0.019	0.007	0.09	0.009	0.002	0.077	0.028	Supported

indPC—PC-RA-UI, indPS—PS-RA-UI, indTAC—TAC-TTF-UI, indTEC—TEC-TTF-UI, indGS—GFS-TTF-U

Users’ perception of the technical characteristics of DCEP and their perception of payment requirements would influence their willingness to adopt DCEP by influencing their perception of technology-task fit. Government support influenced users’ willingness to adopt DCEP by influencing their perception of technology-task fit. Although the perceived security of the DCEP and privacy concerns of the original payment methods positively affected users’ understanding of the relative advantage of DCEP, the mediating effect of the relative advantage was not supported because the relative advantage exhibited no significant effect on the willingness to use DCEP in this study.

## Discussions

### Key findings

The motivation for introducing CBDC varies by country. Payment efficiency and inclusive finance are the primary motivations for CBDC in emerging countries, whereas developed countries pay greater attention to payment and financial security. Some studies have focused on payment-related motivations but have not considered those related to social, security, and monetary policy (Singh et al. 2022). This study discusses users’ willingness to adopt DCEP from the perspectives of payment efficiency and security and hopes to make theoretical and practical contributions to the promotion of DCEP.

Mobile payment methods have brought great benefits, but there are also various drawbacks, such as spam, malware, hacker attacks caused by data theft problems, and financial loss problems due to online fraud. Meanwhile, anonymity, fuzziness, rapidity, and lack of supervision management are unique dark sides of mobile payments (Mogaji and Nguyen 2022). Numerous media reports and academic studies focusing on the issue of privacy and taking it as a concerning issue for network users, and our results reveal that DCEP’s safety awareness and privacy concerns regarding the original payment method can affect the perceived relative advantage of DCEP. However, this relative advantage is insufficient to encourage users to use DCEP. One explanation is that other factors also hinder innovation in the diffusion process. For example, in our model, switching costs have a significant negative impact on DCEP adoption intention, and users reveal uncertainty regarding DCEP. Futhermore, online users may experience privacy disclosure fatigue. Most consumers learn about the risk of privacy disclosure through relevant news reports, notices, warnings, and suggestions to enterprises, but they will not experience subsequent losses (Chen et al. 2021). Simultaneously, DCEP will face significant data security challenges (Bao 2022).

The CBDC offers the highest level of privacy protection among existing electronic payment tools. It is positioned as an alternative to public services rather than an instrument to collect transaction data for commercial interests, which can even restrict commercial institutions from obtaining personal information from users. Third-party payment companies usually provide payment services at a very low price or for free because their business model is to sell customers’ transaction data to retailers. User data leaks alert consumers to be cautious about adopting new payment technologies (Lan 2021).

The influence of government support on users’ willingness to use DCEP was significantly mediated by the degree of technical task fitting. Compared with the relative advantages of risk and safety, the technology-task fitting degree in the payment context significantly positively impacts DCEP's adoption intention. We can match technical characteristics with task characteristics to improve user intentions. Regarding task-technology fit, government support can promote the application of related services. From a strategic point of view, service providers need to better understand how to design and configure DCEP solutions uniquely for corresponding payment scenarios, considering both their capabilities and the problems they can solve for users (Toufaily et al. 2021). Government support also needs to consider the business logic of technology and the impact of payment services on user culture to significantly impact the migration of payment behavior (Xi and Ng 2020). Based on the capability of technical characteristics, task-technology fitting can be used to increase and adjust support services to promote the use of DCEP services and improve individuals’ awareness of the tasks and diversity that the service can meet, thus facilitating users’ switch from the original payment method to DCEP.

Switching cost has a significantly negative impact on users’ willingness to use DCEP, but technology-task fitting has a more significant effect than that for switching cost. In the user migration process, we need to consider the cost of losing users’ interests; that is, when users switch service suppliers, they will lose the rewards and benefits of loyalty. Simultaneously, if there is a relevant contract between the original service provider and users, the cost of loss of interest also includes the potential loss caused by the breach of the contract. Innovative technologies should attempt to reduce the uncertainty cost of DCEP and seek opportunities to build user-friendly relationships as soon as possible. Central banks are responsible for promoting and certifying secure payment options and must ensure the confidence of households, businesses, and other institutions when they introduce secure payment options (Auer et al. 2022).

### Theoretical implications

#### More attention to the specific usage context of DCEP

Previous studies on service adoption and usage have predominantly focused on a single service, considering users’ perceptions of individual products or services or investigating them as a whole without considering the impact of alternative products (Shen et al. 2021). According to the research findings, the technology-task fitting degree is influenced by the technical characteristics of DCEP, and its corresponding task characteristics significantly affect users’ willingness to use DCEP. CBDCs in different countries also require design schemes based on technologies, national conditions, and design purposes.This continuous, ecological, technological, and detailed innovation is easily ignored. Our study combined the PPM framework with the TTF theory and supplemented government support to deepen our understanding of the user behavior of products with similar functions. The study is based on users’ intentions and the actual use of DCEP, which is supported by a wide range of information technologies. This paper may throw some light on the analysis of switching intentions between DCEP and other information system services for information technology experts in banks and other enterprises.

DCEP cannot thoroughly replace any payment methods, indicating that it is more essential to enhance the advantages of DCEP targeting at particular contexts. For individuals, DCEP can maximize the strength of fait money in micropayments. Users can obtain the freedom and speed of digital transaction within an amount limit of trading volume, which is especially adapted for anonymous micropayment trading contexts that do not require high trust between both sides of the transaction. For enterprises, DCEP has the authority of national endorsement, especially in international trade contexts such as the Belt and Road, which is conducive to the development of e-commerce. In global e-commerce and corporate payments, DCEP can exert the advantage of government support to ensure that the transaction process is more convenient and friendly (Wang et al. 2022a, b). Regarding promotions, users with a higher sense of national identity are more likely to use DCEP (Wu et al. 2022). Owing to security concerns and economic benefits, users with less social experience tend to trust new payment tools (Ključnikov et al. 2020), but CBDCs need to design their figures and enhance their popular identity to stimulate soft trust among users (Tronnier et al. 2022; Radic et al. 2022).

#### Supplements TTF theory and PPM framework in the privacy paradox

This study complements and discusses the context of the PPM framework and TTF theory from personal data privacy and security perspectives. Our study attempts to explain users’ adoption behavior toward DCEP by relevant advantages of fintech risks and security. Existing research based on PPM considers mature technologies as the research object, ignoring the difference between the embryonic and developmental stages of innovative technologies. Meanwhile, we take task-technology fitting theory as the driving factor rather than just considering the technical characteristics of DCEP. The controllable anonymity of DCEP is a trade-off between the public’s demand for reasonable privacy protection and the reduction of illegal transaction risks. The transaction data generated is disclosed to the central bank, and relevant data analysis is conducted anonymously. Compared with bank cards, WeChat Pay, Alipay, and other payment tools that require real-name bank account binding, a unique identifier of an individual can be a DCEP account with additional information for upgrading their digital wallet to achieve a balance between privacy and flexibility.

In payment task-technology fitting, government support can enhance people’s trust in payment technology. Government endorsement and support can improve the privacy paradox of users during the payment process. Users tend to focus on immediate interests rather than potential risks when disclosing transaction data to obtain payment services. However, the technology-task fitting theory only considers the direct interests of users, which means the satisfaction of payment needs, ignoring the lack of traceability and privacy security of payment technology. Although DCEP, cash, third-party payment, and other payment methods are committed to solving payment needs in the transaction process, the definition of DCEP is fundamentally different from the other methods, which provides an extension of the research scenario for the PPM framework.

#### Advantages of DCEP originate from the user experience

The central bank’s digital currency benefits different entities and brings about potential risks and complex policy issues. For example, calculating interest may affect payment systems and financial stability differently. However, with the impact of disruptive financial technologies, such as private digital currency, the large-scale adoption of new payment instruments is inevitable.

While discussing and publicizing DCEP, we should also pay attention to China’s payment culture and the background of existing payment methods. Although privacy and security are critical, if users cannot turn them into perceived relative advantages, the promotion of DCEP will also be limited. Since DCEP has adopted the no-service charge operation mode, it is worth discussing whether it needs specific business logic to improve users’ sense of participation and enjoyment.

### Practical implications

#### The popularization of DCEP should excavate the usage context for DCEP

There remains inadequate research in understanding users' payment needs, integrating users' perception of technology-task fit into service functions, and further closely combining related technologies with users' requirements to realize various information service functions. Future research should explore more payment scenarios to break the time and space constraints in the payment process, emphasize the payment context, and grasp the technical characteristics of DCEP. Considering the characteristics of various groups, the pilot will further enhance people’s understanding of DCEP, reduce users’ learning costs of DCEP, and create the relative advantages of DCEP when promoting users’ continuous understanding of DCEP, which still need further exploration.

Tax and CBDC interest rates are still policies that need to be reckoned with to promote the adoption of CBDC, so as to reduce transaction costs and formalize the economy to provide better social protection for the labor force, thus, bringing social welfare benefits. Rapid economic growth and the expanding middle-class population of emerging economies, such as China and India, require the simultaneous improvement of financial infrastructure which is significant for financial inclusion (Allen et al. 2022).

#### DCEP also needs to strengthen the smart and ultimate payment scenario services

Applications such as hard wallets, security chip technologies, wearable devices, and the Internet of things enables us to explore and develop new application scenarios to enhance the universality of digital currency, which releases many messages that deserves our studies. China’s market and people have become path-dependent regarding existing electronic payments. The legal digital currency still needs to strengthen cooperation with the current payment system and financial institutions to form an online and offline service model that improves the adaptability of the scenario and its market acceptance in a short period.

#### DCEP should seek a more prudent, safe, and effective construction mechanism

The payment market has experienced continuous challenges and opportunities in recent decades, from integration and competition to regulation. A well-functioning payment infrastructure is essential for improving the efficiency of financial markets and the entire financial system, enhancing consumer information, and promoting economic interaction and trade in goods and services. Unsafe and inefficient payment systems may hinder the effective transfer of funds between individuals and economic actors. For example, in China, although small and medium-sized enterprises are an essential part of the global economy, their credit risk assessment is complicated and expensive for banks because of the lack of reliable data (Kou et al. 2021b). The distribution of financial data is usually complex, and the DCEP enables the establishment, analysis, and detection of anomalies in large-scale financial datasets under the premise of security (Li et al. 2021). Mature electronic retail payment tools can increase trade and household consumption and produce positive macroeconomic effects (Zhang et al. 2019). The digital currency of the central bank can meet public policy objectives such as inclusive finance, security, and consumer protection and ensure the privacy of payments that the private sector cannot guarantee.

The influence of DCEP on payment efficiency is reflected in the payment field and the process of national informatization. It follows the transformation opportunity of the real economy brought about by digital technology from single-point coordination to intelligent, wide coverage, and low-delay regional coordination, which is of great significance for the credibility of the inclusive financial industry and Internet of Everything. The deployment of the fintech application layer in financial and consumer systems is of profound significance to the information and value-added data industries of cross-industry data fusion.

## Conclusion

This study expands our understanding of the willingness to switch between digital payment instruments. Our research finds that users’ task-technology fit perception has a significantly positive impact on switching intention. Switching cost has a significantly negative effect on migration intention. Compared to the original payment method, users’ perception of DCEP’s relative advantage in fintech risk has no significant impact. These research results confirm the critical role of task-technology fit on users’ switching intentions in payment scenarios and provide implications for the promotion of DCEP. Overall, these findings suggest that privacy concerns, security perception, adaption between requirements and technology, and government support at the organizational level are related to users’ adoption behavior and provide a starting point for further study on fintech payment.

China's social environment and residents' payment habits, age structure, security, and other heterogeneous needs determine the long-term coexistence of DCEP and other payment methods. The design of DCEP is to facilitate the application of online and offline scenarios, meet the differentiated needs of users with multi-subjects, multi-level, multi-categories, and multi-forms, and avoid the use barriers caused by the digital divide. Further studies on digital public service requirements and realistic requirements of specific groups are necessary, along with the popularization and application of DCEP with a more environmentally friendly DCEP initiative (Howard and Rose 2019; Kou et al. 2022).

China is in a leading position in terms of the CBDC pilot scale and the application scenario’s richness. However, it also faces challenges involving public awareness, legal supervision, security, and theoretical research. At the application level, it is recommended to (1) expand the pilot scenarios and scale to improve financial inclusion based on blockchain and smart contract technology, (2) take CBDC’s advanced digital technology and data market advantages, (3) achieve programmability by loading smart contracts that do not affect currency functions and (4) establish a trust mechanism for currency governance in the meta-universe space. For financial institutions in other countries, the coexistence and application of digital assets with monetary-like characteristics, such as stable currency and CBDC, require further discussion (Bao 2022).

## Data Availability

Data is available upon request.

## Abbreviations

CBDC: Central bank digital currency
DCEP: Digital currency electronic payment
PPM: Pull-pull-mooring
TTF: Task-technology fit
